# Conflict of Interest in Peer-Reviewed Medical Journals: The World Association of Medical Editors Position on a Challenging Problem

**DOI:** 10.4103/0975-1483.63143

**Published:** 2010

**Authors:** LE Ferris, RH Fletcher

**Affiliations:** *Dalla Lana School of Public Health, University of Toronto; Clinical Epidemiology Unit, Sunnybrook Health Sciences Centre, Toronto, Ontario, NADA; Chair, WAME Ethics Committee, USA*; 1 *Harvard Medical School, Boston MA; Chair, WAME Policy Committee, USA.; Chair, WAME Policy Committee, E-mail: lef@ices.on.ca*

Conflict of interest in medical publishing exists when a participant’s private interests compete with his or her responsibilities to the scientific community, readers and society. While conflict of interest is common, it reaches the level of concern when ‘a reasonable observer might wonder if the individual’s behavior or judgment was motivated by his or her competing interests’.[1] Having a competing interest does not, in itself, imply wrongdoing. However, it can undermine the credibility of research results and damage public trust in medical journals.

In recent years, the extent of conflict of interest in medical journal articles has been increasingly recognized. Medical journals and the popular media have published numerous examples of competing interests that seemed to have biased published reports.[2–4] Organizations have expressed concern for the effects of conflicts of interest on research,[5] publication[1,6,7] teaching[8] and continuing medical and nursing education.[9]

The World Association of Medical Editors (WAME) is one of the institutions engaged in this discussion. WAME was established in 1995[10,11] to facilitate worldwide cooperation and communication among editors of peer-reviewed journals, improve editorial standards and promote professionalism in medical editing.[12] Membership in WAME is open to all editors of peer-reviewed biomedical journals worldwide; small journals in resource-poor countries are well represented. As of December 2009, WAME had 1595 individual members representing 965 journals in 92 countries. WAME has broad participation as there are no dues and WAME activities are largely carried out through the member list serve and the member password protected website.

In March 2009, WAME released an updated policy statement, ‘Conflict of Interest in Peer-Reviewed Medical Journals’.[1] It details the issues WAME believes journals should address when establishing their own policies for conflict of interest. The editors of this journal thought that the issues were important enough to share with its readers. A summary of the statement is presented in Table 1 and the full statement[1] can be found on WAME’s website.[12]

**Table 1:** Summary of key elements for peer-reviewed medical journal’s conflict of interest policies

Element	Key aspects	Comments
Definition and Scope	A clear definition the journal uses as to what is conflict of interest and who is captured in the definition.	Sample definition: Conflict of interest exists when a participant in the publication process (author, peer reviewer or editor) has a competing interest that could unduly influence (or be reasonably seen to do so) his or her responsibilities in the publication process (submission of manuscripts, peer review, editorial decisions and communication between authors, reviewers and editors).
Types of Competing Interests	A clear statement of examples of the types of competing interests (and their definitions) the journal says must be declared. Should include the following as examples but there could be others: Financial ties Academic commitments Personal relationships Political or religious beliefs Institutional affiliations	There is a need to consider a wide range of competing interests (and a recognition that they can coexist) which the individual assess as to whether they unduly influence (or be reasonably seen to do so) his or her responsibilities in the publication process. Examples and definitions of what competing interests should be declared needs to be articulated with Journals moving beyond just financial conflict of interest.
Declaring Conflict of interests	Clear statements on (a) what is to be declared, when and to whom; (b) format for declaration; (c) a journal’s role in asking additional questions or seeking clarification about disclosures and (d) consequences for failing to disclose before or after publication.	Journals rely on disclosure about the facts because *routine* monitoring or investigation is not possible. This creates a particular onus on the declarer to report carefully and comprehensively. It also means that journals should ask about conflict of interest in such a way that there will be a high likelihood of reporting relevant conflict of interest.
Managing conflict of interests	A clear statement on how conflict of interest will be managed by the journal, including the position that all relevant conflict of interest disclosures (or the declaration of no conflict of interest) will be published with the article and clarity about what conflict of interest situations will result in a manuscript not being considered.	Journals use various rules about how they will deal with conflict of interest and conflict of interest disclosures and these need to be made known to all those involved in the publication process.
		
		
		
		
		

## HOW DOES THIS STATEMENT DIFFER FROM EARLIER CONFLICT OF INTEREST STATEMENTS?

First, WAME expands the scope of competing interests. Other statements have been concerned almost exclusively with conflicts of interest related to financial ties to industry - companies that sell healthcare products. The assumption is that financial incentives are especially powerful and are not readily recognized without special efforts to make them apparent. WAME has extended the concept of financial conflict of interest to include the effects of clinical income. For example, physicians who earn their livelihood by reading mammograms or performing colonoscopies may be biased in favor of these technologies. WAME has also included non-financial conflicts of interest (or the appearance of one) related to scholarly commitment: ‘intellectual passion’, (the tendency to favor positions that one has already espoused or perhaps even established); personal relationships (the tendency to judge the works of friends/colleagues or competitors/foes differently because of the relationship); political or religious beliefs (the tendency to favor or reject positions because it affirms or challenges one’s political or religious beliefs) and institutional affiliations (the tendency to favor or reject results of research because of one’s institutional affiliations).

Second, WAME did not prescribe a universal standard for when meaningful conflict of interest exists. Rather, it defined and recommended elements of conflict of interest policies and encouraged journals to establish their own standards. WAME left operational definitions and standards on the basic issues to member journals, recognizing that journals exist in very different contexts across the globe, standards for conflict of interest are evolving and some journals already have well-established policies and standards. WAME does not presume to judge which conflicts require action and what the appropriate action may be, although its policy does offer factors to consider.[1] Obviously, excessive concern for these and more comprehensive lists of possible competing interests could paralyze the peer review and publication process and is not feasible. Editors must make judgments as to the strength of the conflict, but to do so must have uncensored information. Similarly, readers need transparency about conflicts, and therefore editors should publish with every article all relevant author disclosures.[1]

Third, WAME confirms the seriousness of failure to disclose conflict of interest by indicating that editors have a responsibility for investigating, and if relevant acting, if competing interests surface after a manuscript is submitted or published. The intent is that allegations of failure to declare conflicts of interest must be taken seriously by journals.

Finally, WAME has addressed in a single statement the conflicts of interests threatening all participants in the research and publication continuum, including authors, peer reviewers and editors. Conflicts between editors and journal owners, which might affect both the accuracy of articles and the credibility of journals, have been addressed in another WAME policy statement.[13]

## WHAT CAN BE DONE ABOUT CONFLICT OF INTEREST IN MEDICAL JOURNALS?

Conflicts of interest cannot be eliminated altogether but it can be managed so that it has the smallest possible effects on journal content and credibility. The backbone of managing conflicts of interest is full written disclosure; without it, nothing else is possible. Currently, authors may not reveal all of their competing interests and even if they do, journals too often do not publish them,[14] so there is plenty of room for improvement. Even so, disclosure alone is an imperfect remedy; editors still must determine whether a conflict has sufficient potential to impair an individual’s objectivity such that the article should not be published. Even more work may be needed on reviewers’ and editors’ competing interests, given their critical role as gatekeepers for the medical literature.

No statement will solve the conflict of interest problem, nor will it ever be solved altogether. As understanding of the problem and its management evolves, journals should be given latitude to establish their own standards, matching their policies to the best standards of their discipline and culture. WAME believes journals should make these policies readily accessible to everyone. All of us—editors, authors, reviewers and readers—should be paying more attention to conflict of interest than we have been. We hope this statement serves that purpose.
